# Innate immune responses to three doses of the BNT162b2 mRNA SARS-CoV-2 vaccine

**DOI:** 10.3389/fimmu.2022.947320

**Published:** 2022-08-22

**Authors:** Marina Saresella, Federica Piancone, Ivana Marventano, Ambra Hernis, Daria Trabattoni, Mattia Invernizzi, Francesca La Rosa, Mario Clerici

**Affiliations:** ^1^ IRCCS Fondazione Don Carlo Gnocchi, Milan, Italy; ^2^ Departments of Biomedical and Clinical Sciences L. Sacco, University of Milan, Milan, Italy; ^3^ Environmental Science and Policy, University of Milan, Milan, Italy; ^4^ Pathophysiology and Transplantation, University of Milan, Milan, Italy

**Keywords:** mRNA vaccine, innate immunity, natural killer, KIR, IFN-γ

## Abstract

To explore the effects of SARS-CoV-2-mRNA vaccines on innate immune responses we enrolled 58 individuals who received 3 doses of the BNT162b2 vaccine in a longitudinal study; 45 of these individuals had never been SARS-CoV-2 infected. Results showed that vaccination significantly increased: 1) classical and intermediate inflammatory monocytes, 2) CD56^bright^, CD56^dim^, and CD56^dim^/CD16^dim^ NK cells, and 3) IFN-γ+ ;production as well as perforin and granzyme content by NK cells. Vaccination also reduced expression of the NK inhibitory receptor ILT-2, increasing that of the stimulatory molecule 2DS2. These effects were long-lasting and were boosted by every vaccine dose. Notably, ILT-2 expressing NK cells were reduced even more robustly in COVID-19-recovereed vaccines. BNT162b1 mRNA vaccine is known to induce potent adaptive immune responses; results herein show its ability to modulate innate immune responses as well, offering further support to the indication to proceed with worldwide vaccination efforts to end the SARS-CoV-2 pandemic.

## Introduction

The severe acute respiratory syndrome coronavirus 2 (SARS-CoV-2) is responsible for coronavirus disease 2019 (COVID-19), a pathology characterized by an extremely high infectivity but a relatively low pathogenicity. Thus, while the majority of patients experience mild symptoms, a minority develops a multi organ condition that, in the worst cases, results in a severe and potentially lethal acute respiratory distress syndrome (ARDS) (1). SARS-CoV-2 took the world by storm in early 2020 and has provoked a worldwide pandemic that, as of today, has claimed more than 6 million of lives (2). Monoclonal antibodies and antivirals have been developed that can modulate the severity of COVID-19 (3, 4). Most importantly, though, an array of highly efficient vaccines has been created within less than a year; the extensive use of these vaccines has allowed the containment of SARS-CoV-2 infection and has changed the natural history of the disease, greatly reducing infections, severe cases and deaths (5).

Amongst SARS-CoV-2-specific vaccines, lipid nanoparticle (LNP)-formulated mRNA vaccines are standing out due to their novelty and their extremely high protective efficacy. The BNT162b1 vaccine (BioNTech-Pfizer), in particular, contains mRNA that encodes the receptor-binding domain (RBD) of the SARS-CoV-2 spike protein. This vaccine results in a >95% efficacy in preventing severe COVID-19 (6) as a result of its ability to potently elicit specific adaptive immune responses, as demonstrated by different studies in humans (7–11). Notably, in contrast with the numerous studies on the effects of mRNA vaccines on adaptive immunity, their effect on innate immune responses has been barely analysed. Recent results showed that BNT162b1 vaccination results in an enhancement of myeloid cell-mediated anti-viral responses that are enhanced by secondary immunization (12). These results led to the creation of a model in which ‘cytokine feedback’ upregulates innate immune response after vaccine boosters. This model does not preclude the possible presence of other mechanisms, including the induction by vaccines of “trained immunity”, a condition associated with epigenetic changes resulting in the generation of immunological memory in innate immune cells (13, 14).

We verified the possible BNT162b2 vaccine-mediated long-term modulation of innate immunity by analysing monocyte and natural killer subsets in peripheral blood, as well as cytokine production and cytotoxicity after spike protein stimulation. Analyses were performed at baseline, after initial inoculation, and after a first and a second booster vaccine dose in a group of individuals who had never been SARS-CoV-2-infected. The inclusion in the group of analysed individuals of a subset of subject that recovered from COVID-19 disease allowed the comparison of vaccinal responses in these two immunologically different scenarios.

## Materials and methods

### Study design

**Figure 1:**
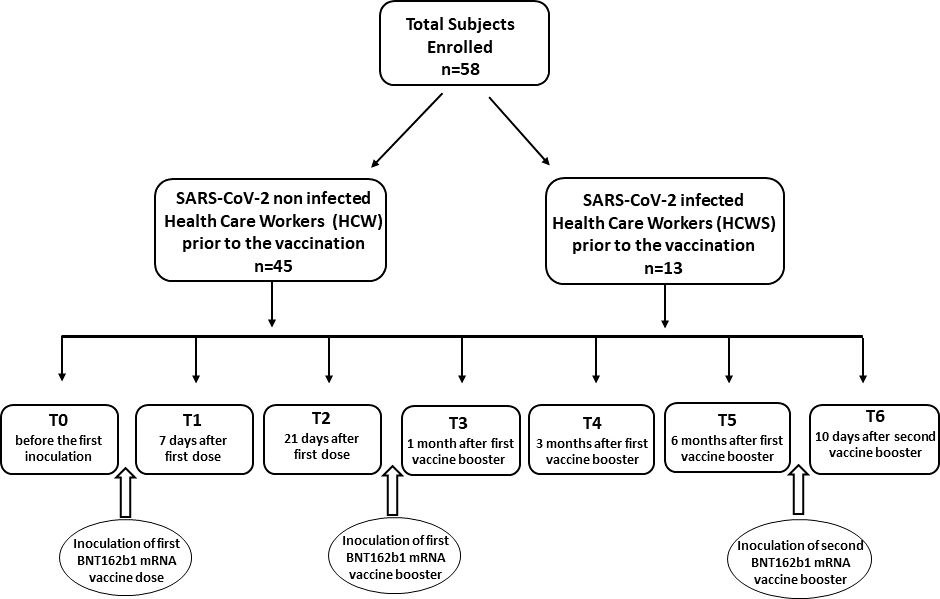
Synoptic representation of the study design with timing. The enrolled subjects involved in the study were Healthy Care Workers who had not (HCW) or had (HCWS) been SARS-CoV-2-infected prior to the initiation of the vaccine protocol.

A longitudinal study to define the long term innate immune response to the BNT162b1 vaccine, which encodes the receptor-binding domain (RBD) (subunit 1) of the SARS-CoV-2 spike protein, was performed in fifty-eight Health Care Workers. Forty-five of these Health Care Workers had never been SARS-CoV-2-infected before receiving the first immunization (HCW); thirteen other Health Care Workers did suffer from mild COVID-19 infection and recovered without showing any lasting effects (HCWS) (**Table 1**). Immune responses were monitored at different time points: baseline (immediately before the first inoculation; T0), 7 (T1) and 21 (T2) days after initial inoculation, one (T3), three (T4) and six (T5) months after the first vaccine booster, and, finally, ten days after the second vaccine booster (T6). Study design is summarized in **Figure 1**. Immunological analyses focused on the immunophenotype of monocytes and Natural Killer cells (NK). Intracellular IFN-γ- and TNF- producing NK and monocytes were analyzed after stimulating whole PBMC with the recombinant 2019-nCoV Spike protein (S1 subunit; spike) (see below).

**Table 1:** Demographic characteristics of the enrolled individuals.

	HCW	HCWS
** N** ** **	**45**	**13**
**Age (years) median** **[interquartile range]**	**45** **(34-56)**	**48** **(45-52)**
**Sex M/F**	**16/29**	**5/8**

N, number; M, male; F, female; HCW, Healthy Care Workers who had not been SARS-CoV-2-infected prior to the initiation of the vaccine protocol, HCWS, Healthy Care Workers who had been SARS-CoV-2-infected prior to the initiation of the vaccine protocol.

The study was approved by the local ethics committee (Comitato Etico – IRCCS Fondazione Don Gnocchi, Milano) and all the individuals enrolled in the study provided written informed consent.

### Blood sample collection

Whole blood was collected in vacutainer tubes containing ethylenediamine tetra-acetic acid (EDTA) (Becton Dickinson & Co., Rutherford, NJ, USA). Cell counts were performed using an XN-1000 Sysmex hematology analyzer (Dasit Group Italy) and viability was evaluated using the automated cell counter ADAM-MC (Digital Bio, NanoEnTek Inc., Korea) after red cell lysis. Peripheral Blood Mononuclear cells (PBMC) were separated on lymphocyte separation medium (Organon Teknika Corp., Durham, NC) and washed twice in PBS; viable leukocytes and count were determined as above.

### *In vitro* PBMC stimulation

PBMCs were counted and resuspended at 2 million/ml in complete RPMI supplemented with 10% FBS and antibiotics (Invitrogen, Ltd., Paisley, UK). Cells were incubated overnight at 37 °C in a CO_2_ incubator. The next morning, cells were resuspended and 2 ml of cell suspension was added to wells of a 12-well tissue culture plate. Each analysis was performed in duplicate. Intracellular IFN-γ, TNF, perforin and granzyme content was analysed by flow-cytometry in cells that had been cultured in medium alone (unstimulated) or had been stimulated with the recombinant 2019-nCoV Spike protein (S1 subunit) (Novateinbio Biosciences, Woburn, MA, USA) at a concentration of 1 μg/ml. Brefeldin A (Sigma)(10 μg/mL) was added to the cells cultures prior to a 24 hour incubation at 37 °C in a CO_2_ incubator. PBMC were collected and prepared for Flow-cytometry analysis.

### Immunofluorescent staining and analysis by flow-cytometry

Immunophenotypic analyses of monocytes and NK were performed on 600 μl of EDTA peripheral blood incubated for 30 minutes at 4° C with fluorochrome-labeled monoclonal antibodies. Erythrocyte lysis was obtained with the Immuno-Prep Epics Kit and Q-Prep Work Station (Beckman-Coulter Brea, CA, USA). To analyse NK subpopulations 20.000 events were acquired and gated on Forward and Side scatter properties for lymphocytes, and on CD3-CD19-CD14- and Side scatter properties to exclude T, B and monocytes; the remaining triple-negative cells were analyzed in a CD56 versus CD16 dot plot to identify the natural killer (NK) cell subsets, CD56^bright^CD16−, CD56^bright^CD16^dim^, CD56^dim^CD16^bright^, CD56^dim^CD16^dim^, CD56^dim^CD16− and CD56−CD16^bright^. The expression of KIR receptors was performed on NK subsets. To analyse monocytes subpopulations 20.000 events were acquired and gated on a SSC/CD14 scatter plot and on a CD14+; another three gates were set on CD14/CD16 scatter plot to distinguish classical, (CD14^bright^/CD16^-^), intermediate (CD14^dim^/CD16^dim^), and non-classical monocytes (CD14^dim/neg^/CD16^bright^).

Immune response of innate cells against SPIKE Sars-Cov2 protein (S1) was measured using an intracellular cytokine staining assay. Briefly, unstimulated and stimulated PBMC were washed in PBS, split in two tubes, and stained with anti-CD3, -CD56, -CD14 and –CD107 mAb for 30 minutes at 4°C in the dark. PBMC were then washed and the intracellular staining of IFN-γ, TNF, perforin and granzymes was obtained using the FIX & PERM kit (Invitrogen-Caltag Lab Carlsbad, CA, USA). For each analysis, 20.000 events were acquired and gated on Forward (FSC) and Side scatter (SSC) properties for lymphocytes and on CD3-CD14-CD56+ to define NK; the monocyte gate was designed on CD14+/SSC. Intracellular IFN-γ and TNF were analyzed in NK and monocytes; perforin and granzymes were analyzed in NK cells alone.

Analyses were performed using a Beckman-Coulter GALLIOS flow cytometer equipped with a 22 mW Blue Solid State Diode laser operating at 488 nm and with a 25 mW Red Solid State Diode laser operating at 638 nm and interfaced with Kaluza analysis software. Flow cytometry compensation was performed using the fluorescence minus one (FMO) approach. Briefly all antibody conjugates in the experiment are included except the one that is controlled for. The FMO measures the spread of fluorescence from the other staining parameters into the channel of interest, determining the threshold for positive staining.

### Monoclonal antibodies (mAbs)

The following mAbs were used: anti- CD3 phycoerythrin-cyanine 7 (PE -Cy7) (Mouse IgG1, Clone: UCHT1) (Beckman-Coulter); anti- CD19 PC-7 or FITC (Mouse IgG1, Clone: J3-119) (Beckman-Coulter); anti- CD14 PC- 7 (IgG2a Mouse, clone: RMO52)(Beckman-Coulter); anti-CD16 PE or PE-Cy5 or Alexa-750 (Mouse IgG1, Clone: 3G8) (Beckman Coulter); anti-CD56 PE or PE-Cy5.5 or PC-7 (Mouse IgG1, Clone: N901 (NKH-1) (Beckman Coulter); anti- CD107a FITC (LAMP-1) (Mouse IgG1, kappa, Clone: eBioH4A3) (eBioscience-TermoFisher Waltham, MA USA), anti-Human KIR2DL1/CD158a FITC (Mouse IgG1, Clone: 143211), (R&D Systems, Minneapolis, MN, USA), anti-Human KIR2DS4/CD158i Allophycocyanin (APC) (Mouse IgG2a, Clone: 179315) (R&D Systems); anti-Human ILT-2/CD85j APC (Mouse IgG1, Clone: 292305) (R&D Systems); anti-Human KIR2DS1/CD158h Alexa Fluor 700 (Rabbit IgG, Clone: 1127B) (R&D Systems); anti-KIR2DS2/CD158b Polyclonal Antibody FITC (Rabbit IgG aa39-65) (LSBio, Seattle WA USA); anti-TNF-FITC or PE (MP9-20A4), anti-IFN-γ-PE or FITC (B27) (Invitrogen-Caltag Lab Carlsbad, CA, USA), anti-perforin APC (δ.G9)(m IgG2b), and anti-granzyme-PE (GB11)(m IgG1)(Becton-Dickinson Biosciences, San Jose CA, USA).

### Statistical analysis

Quantitative data were not normally distributed (Shapiro-Wilk test) and are thus summarized as median and Interquartile Range (IQR; 25° and 75° percentile). Comparisons between groups were analyzed to evaluate immunological differences. Kruskal-Wallis analysis of variance was performed for each variable; Bonferroni correction was applied to the results. p values and all tests are two-sided. A P value < 0.05 was considered statistically significant. Data analysis was performed with the Python 3.7.4 libraries: Matplotlib, Statsmodels, Scipy.

## Results

### Modulation of monocyte subsets by multiple doses of the BNT162b1 vaccine

The ability of multiple doses of the BNT162b1 SARS-CoV-2 mRNA vaccine to modulate different monocyte subpopulations was investigated before (baseline)(T0) and after the initial dose of vaccine, as well as after the first and the second vaccine boosters in 45 individuals who had never been infected with SARS-CoV-2. The three main known functional and phenotypical monocyte subpopulations that were analyzed were defined as follows: classical (CD14^bright^), intermediate (CD14^dim^/CD16^dim^), and non-classical (CD14^dim^/CD16^bright^) monocytes

Results showed that multiple doses of vaccine do indeed result in a differential modulation of such subpopulations, resulting in a preferential stimulation of classical and intermediate monocytes. Thus, as compared to baseline (T0), the percentage of: 1) classical monocytes was increased 7 days after the initial dose (T1), one-month (T3) after the first booster, and 10 days (T6) after the second booster (T0 vs. T6 p<0.005); and 2) intermediate monocytes was increased 7 days after priming (T1) and six months (T5) after the first booster dose, and remained increased compared to baseline values at the end of the study period (T6) (T0 vs. T6 p<0.005). In contrast with these results, the percentage of non-classical monocytes was reduced compared to baseline six months (T5) after the first booster dose as well as 10 days (T6) after the second booster dose (T0 vs. T6 p<0.005).

**Figure 2:**
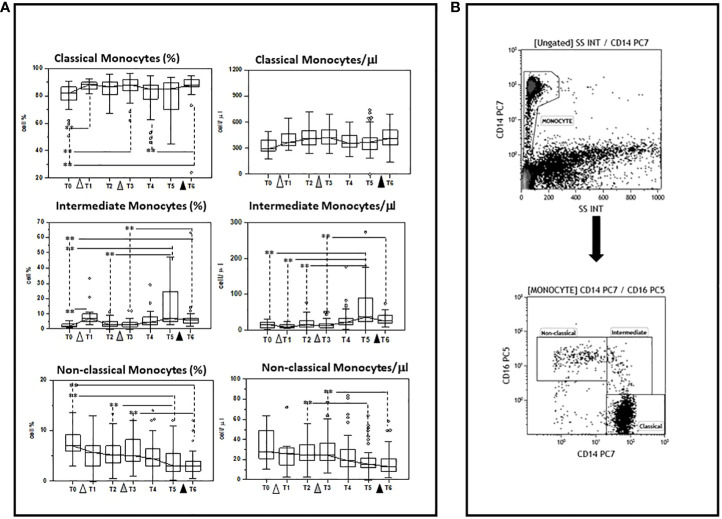
Modulation of monocyte subpopulations by multiple doses of the BNT162b1 vaccine. **(A)** Summary results indicating different monocyte subsets are shown as percentages (medians) in the left panel and as absolute numbers in the right panel. Individuals who had never been SARS-CoV-2-infected and received three doses of the BNT162b1 mRNA vaccine were analysed at different time points: baseline (immediately before the first inoculation (white triangle) (T0), 7 (T1) and 21 (T2) days after initial inoculation, one (T3), three (T4) and six (T5) months after the first vaccine booster (grey triangle), and, finally, ten days after the second vaccine booster (black triangle) (T6). Boxes stretch from the 25th to the 75th percentile. Lines across the boxes indicate the median values. Lines stretching from the boxes indicate extreme values. Statistical significance is shown. **p<0.05 **(B)** Gate strategy to selected monocyte cell subsets: **(A)** the CD14+vs SSC dot plot allows the discrimination of monocyte **(B)** monocyte subsets were defined in the CD14 vs CD16 dot plot as: Classical (CD14^high^CD16^neg^), intermediate (CD14^dim^/CD16^dim^), and non-classical (CD14^dim/neg^/CD16^bright^) monocytes.

The absolute numbers of these subsets followed the same trend: classical and intermediate cells (classical monocytes: T0 vs. T6 p<0.005) were increased, whereas non-classical monocytes were reduced by vaccination, (T0 vs. T6 p<0.005). These results are summarized in **Figure 2** and **Supplementary Tables 1**, **2**.

### Modulation of NK subsets by multiple doses of the BNT162b1 vaccine

We next investigated whether multiple doses of the BNT162b1 vaccine could influence NK subpopulations as well; to this goal we analyzed at the same times indicated above the following subpopulations of cells: CD56 ^bright^, CD56^dim^, CD56^dim^CD16^dim^, CD56^dim^CD16 ^bright^ and CD16 ^bright^.

Also in this case, vaccination resulted in a complex modulation of NK subpopulations. Thus, as compared to baseline (T0) the percentage of: 1) CD56^dim^; 2) CD56 ^bright^, and 3) CD56^dim^CD16^dim^ NK cells was increased at different timepoints and was significantly augmented in the case of CD56 ^bright^ and CD56^dim^CD16^dim^ NK cells when results after the second booster dose were compared to baseline (T0 vs. T6 p<0.005 in both cases). In contrast with these results, the percentage of CD56^dim^CD16 ^bright^ NK cells was significantly reduced at T6 compared to T0 (p<0.005). Finally, no differences were seen in CD16 ^bright^ NK cells at any time point.

**Figure 3:**
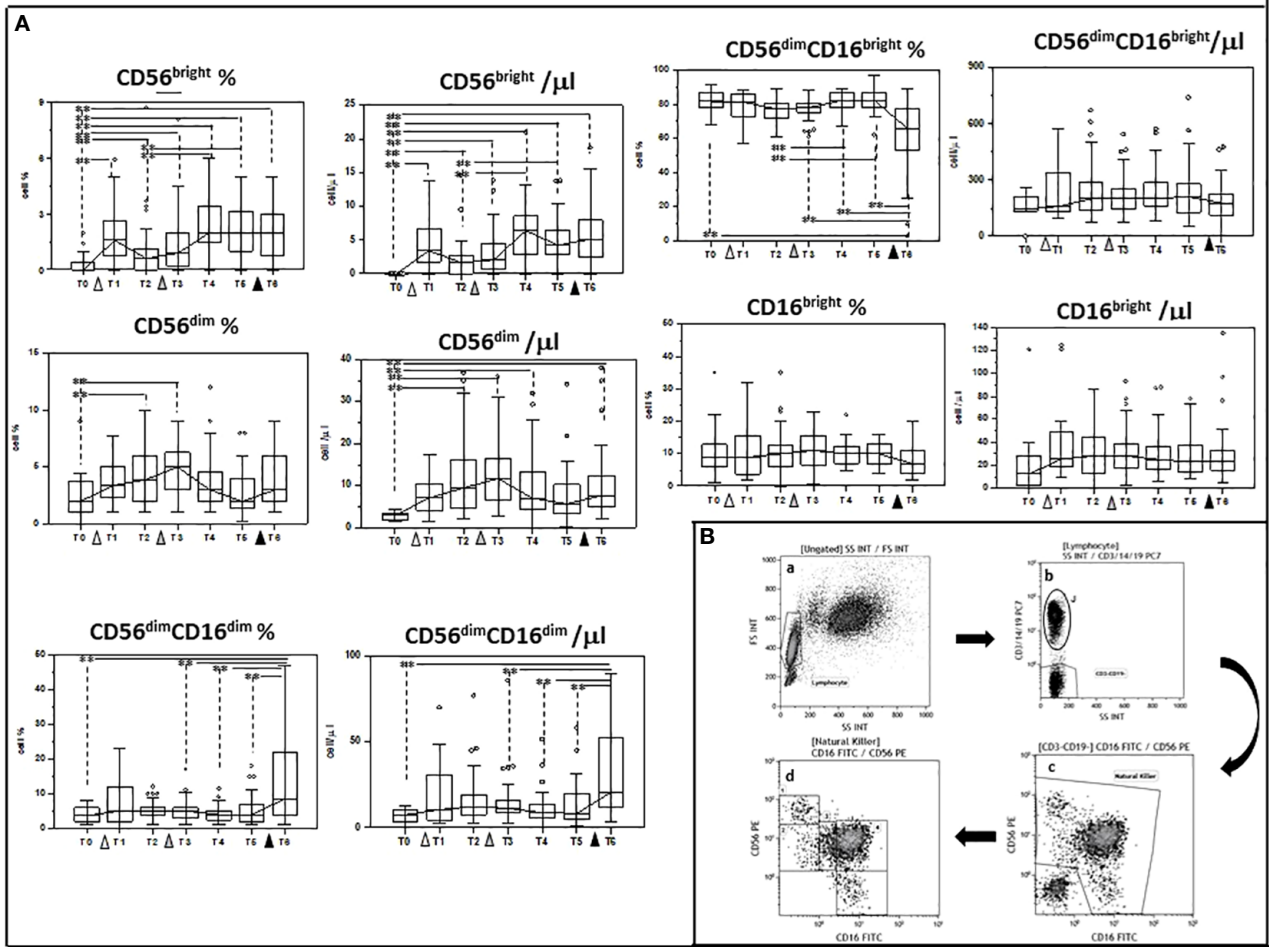
Modulation of NK subpopulations by multiple doses of the BNT162b1 vaccine. **(A)** Summary results indicating different NK cell subsets are shown as percentages (medians) in the left panel and as absolute numbers in the right panel. Individuals who had never been SARS-CoV-2-infected and received three doses of the BNT162b1 mRNA vaccine were analyzed at different time points: baseline (immediately before the first inoculation (white triangle) (T0), 7 (T1) and 21 (T2) days after initial inoculation, one (T3), three (T4) and six (T5) months after the first vaccine booster (grey triangle), and, finally, ten days after the second vaccine booster (black triangle) (T6). Boxes stretch from the 25th to the 75th percentile. Lines across the boxes indicate the median values. Lines stretching from the boxes indicate extreme values. Statistical significance is shown; **p<0,05. **(B)** Gate strategy used to identify NK cell subsets. **(a)** Lymphocyte selected by forward (FS) and side scatter (SS) properties (Gate Lymphocyte). **(b)** The CD3+CD19+ vs. the SS dot plot allows the discrimination of T and B lymphocytes (Gate J); the remaining double negative cells (Gate CD3-CD19-) were analyzed within a CD56 vs. CD16 dot plot **(c)** leading to the identification of NK cells (Gate Natural Killer). **(d)** NK subsets were defined in the CD56 vs CD16 dot plot as: CD56^bright^CD16− (1), CD56^dim^CD16− (2), CD56^dim^CD16^dim^ (3), CD56^dim^CD16^bright^ (4), and CD56−CD16^bright^ (5).

The absolute numbers of these NK cells subsets followed a similar trend, with CD56^dim^, CD56^bright^, and CD56^dim^CD16 ^dim^ being increased (T0 vs. T6 p<0.005 in all cases), and CD56^dim^CD16 ^bright^ showing a trend toward reduction at the end of the study period. These results are shown in **Figure 3** and **Supplementary Tables 1**, **2**.

### Modulation of KIR receptor expression by multiple doses of the BNT162b1 vaccine

**Figure 4:**
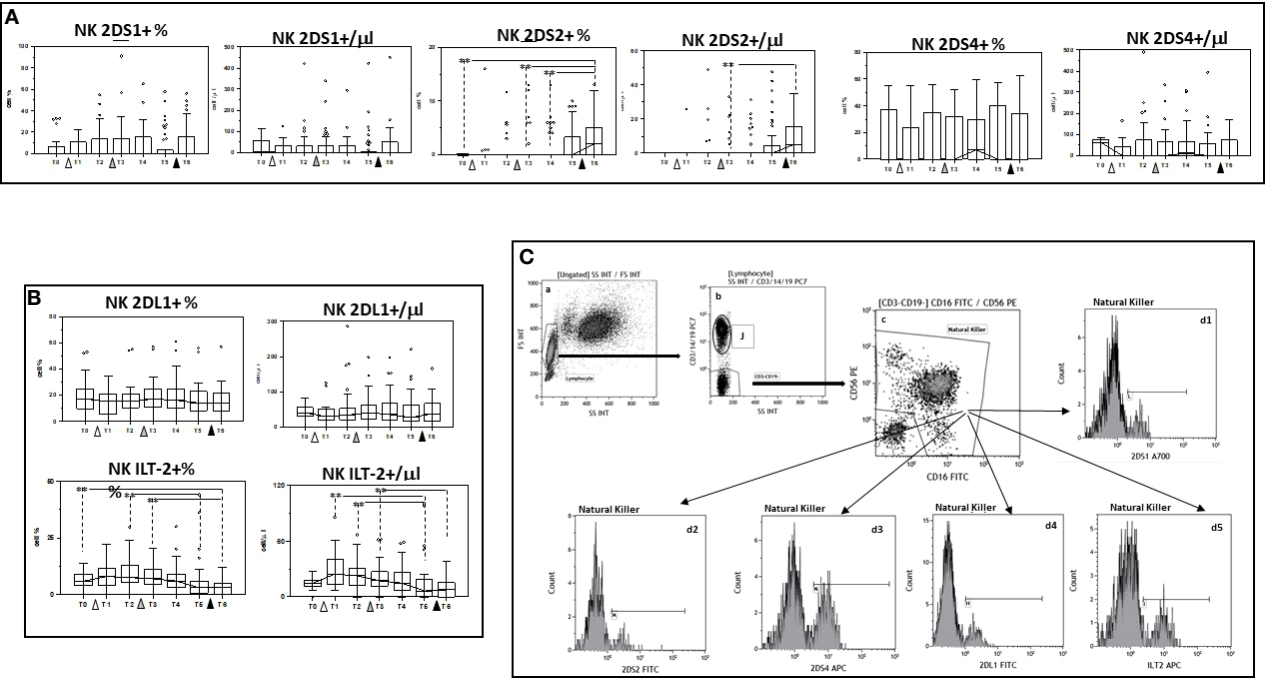
Modulation of NK cells expressing activating and inhibitory receptors by multiple doses of the BNT162b1 vaccine. Summary results indicating percentages and absolute numbers of NK cells expressing the 2DS1, 2DS2 and 2DS4 activating **(A)** or the 2DL1 and ILT-2 inhibitory KIR receptors **(B)**. Individuals who had never been SARS-CoV-2-infected and received three doses of the BNT162b1 mRNA vaccine were analyzed at different time points: baseline (immediately before the first inoculation (white triangle) (T0), 7 (T1) and 21 (T2) days after initial inoculation, one (T3), three (T4) and six (T5) months after the first vaccine booster (grey triangle), and, finally, ten days after the second vaccine booster (black triangle) (T6). Boxes stretch from the 25th to the 75th percentile. Lines across the boxes indicate the median values. Lines stretching from the boxes indicate extreme values. Statistical significance is shown; **p<0,05. **(C)** Gate strategy used to identify NK cell subsets. **(a)** Lymphocyte selected by forward (FS) and side scatter (SS) properties (Gate Lymphocyte). **(b)** The CD3+CD19+ vs. the SS dot plot allows the discrimination of T and B lymphocytes (Gate J); the remaining double negative cells (Gate CD3-CD19-) were analyzed within a CD56 vs. CD16 dot plot **(c)** leading to the identification of NK cells (Gate Natural Killer); (d1-d5) histograms representing the activating (2DS1,2DS2 and 2DS4) and inhibitory (2DL1 and ILT-2) receptors expressed by NK.

The expression of 2DS1, 2DS2, and 2DS4, KIR activating receptors, as well as that of the KIR inhibitory receptors 2DL1 and ILT-2 was analyzed next on NK cells. Results showed that, whereas no significant differences were induced by vaccination in either the percentage or absolute number of NK cells expressing 2DS1 or 2DS4, 2DS2-expressing NK cells were significantly augmented when results obtained at the end of the study period, after three doses of vaccine, were compared to baseline (p<0.005). Vaccination had an opposite effect on inhibitory KIR receptors expression. ILT-2-expressing NK cells, in particular, were significantly reduced by the three doses of vaccine (**Figure 4** and **Supplementary Tables 1**, **2**).

### Cytokine production by spike-stimulated monocytes and NK cells

**Figure 5:**
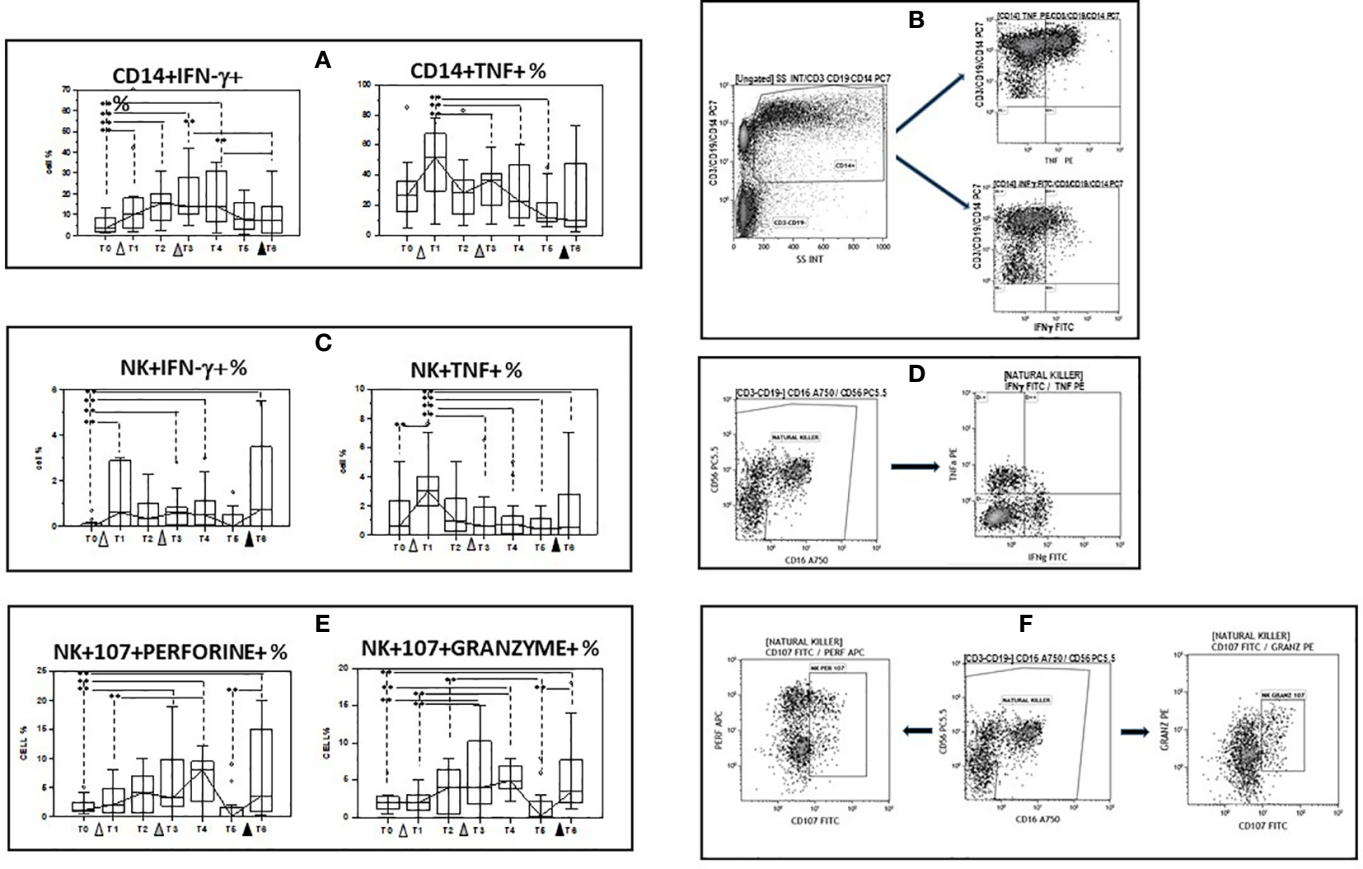
Modulation of cytokine production and lytic and apoptotic molecules expression in innate immune cells by multiple doses of the BNT162b1 vaccine. **(A)** INF-γ and TNF- producing monocytes; **(B)** Gate strategy used to identify cytokine-producing monocytes. The CD14+ vs. the SS dot plot (left) allows the discrimination of monocytes (Gate CD14); cytokine-producing monocytes were identified in the CD14 vs. TNF (upper right) and the CD14 vs. IFN-γ (lower right) dot plots. **(C)** INF-γ and TNF- producing NK cells; **(D)** Gate strategy used to identify NK cells producing cytokines. Double negative cells (Gate CD3-CD19-) were analyzed within a CD56 vs. CD16 dot plot (left) leading to the identification of NK cells Cytokine-producing NK cells were selected in the TNF vs. IFN-γ dot plot (right); **(E)** CD107a+/perforin+ and CD107a+/granzymes+ NK cells. **(F)** Gate strategy used to identify NK cells expressing perforin and granzymes. Double negative cells (Gate CD3-CD19-) were analyzed within a CD56 vs. CD16 dot plot (left) leading to the identification of granzymes- and perforin-producing NK cells in the granzyme vs. CD107 (right) and in the perforin vs. CD107 (right) dot plots. PBMC were stimulated with the SARS-CoV-2 spike protein. Individuals who had never been SARS-CoV-2-infected and received three doses of the BNT162b1 mRNA vaccine were analyzed at different time points: baseline (immediately before the first inoculation (white triangle) (T0), 7 (T1) and 21 (T2) days after initial inoculation, one (T3), three (T4) and six (T5) months after the first vaccine booster (grey triangle), and, finally, ten days after the second vaccine booster (black triangle) (T6). Median values are shown. Boxes stretch from the 25th to the 75th percentile. Lines across the boxes indicate the median values. Lines stretching from the boxes indicate extreme values. Statistical significance is shown, **p<0,05.

Immune responses against pathogens are the sum of the activation of multiple effector mechanisms that are mediated by different cell types, to approach the *in vivo* situation we examined IFN-γ- and TNF-producing monocytes and NK cells after stimulating PBMC with the spike Sars-Cov2 protein (spike). Results obtained upon spike stimulation (background subtracted) showed that the initial dose of vaccine as well as the first booster dose increased the percentages of IFN-γ- producing monocytes and NK cells compared to baseline values. This effect was only observed for IFN-γ- producing NK cells after the second vaccine booster, and these cells were significantly increased at the end of the study period compared to T0 (p<0.05). Percentages of TNF-producing monocyte and NK cells increased after the initial dose of vaccine, but were decreased compared to baseline at the end of the study. (**Figures 5A–D** and **Supplementary Table 3**).

### Perforin- and granzyme- containing and CD107a-expressing NK cells

Secretory lysosomes of natural NK cells contain perforin and granzymes; these proteins mediate NK-cell cytotoxicity as their release results in the induction of target-cell lysis and apoptosis. Lysosome-associated membrane protein (LAMP) 1/CD107a is used as a marker for NK-cell degranulation and cytotoxicity. We analysed CD107-expressing and granzyme- or perforin- containing NK cells in spike-stimulated PBMC. Results obtained upon spike stimulation (background subtracted) showed that vaccination resulted in an increase of both cell types, with booster doses further augmenting both populations. As a consequence, both CD107a+/granzyme and CD107a+/perforin+ NK cells were significantly increased when results at the end of the study (T6) were compared to baseline (p<0.005 in both cases). These results are summarized in **Figures 5E, F** and **Supplementary Table 3**.

### Innate immune responses to the BNT162b1 vaccine in individuals who had or had not been SARS-CoV-2-infected

**Figure 6:**
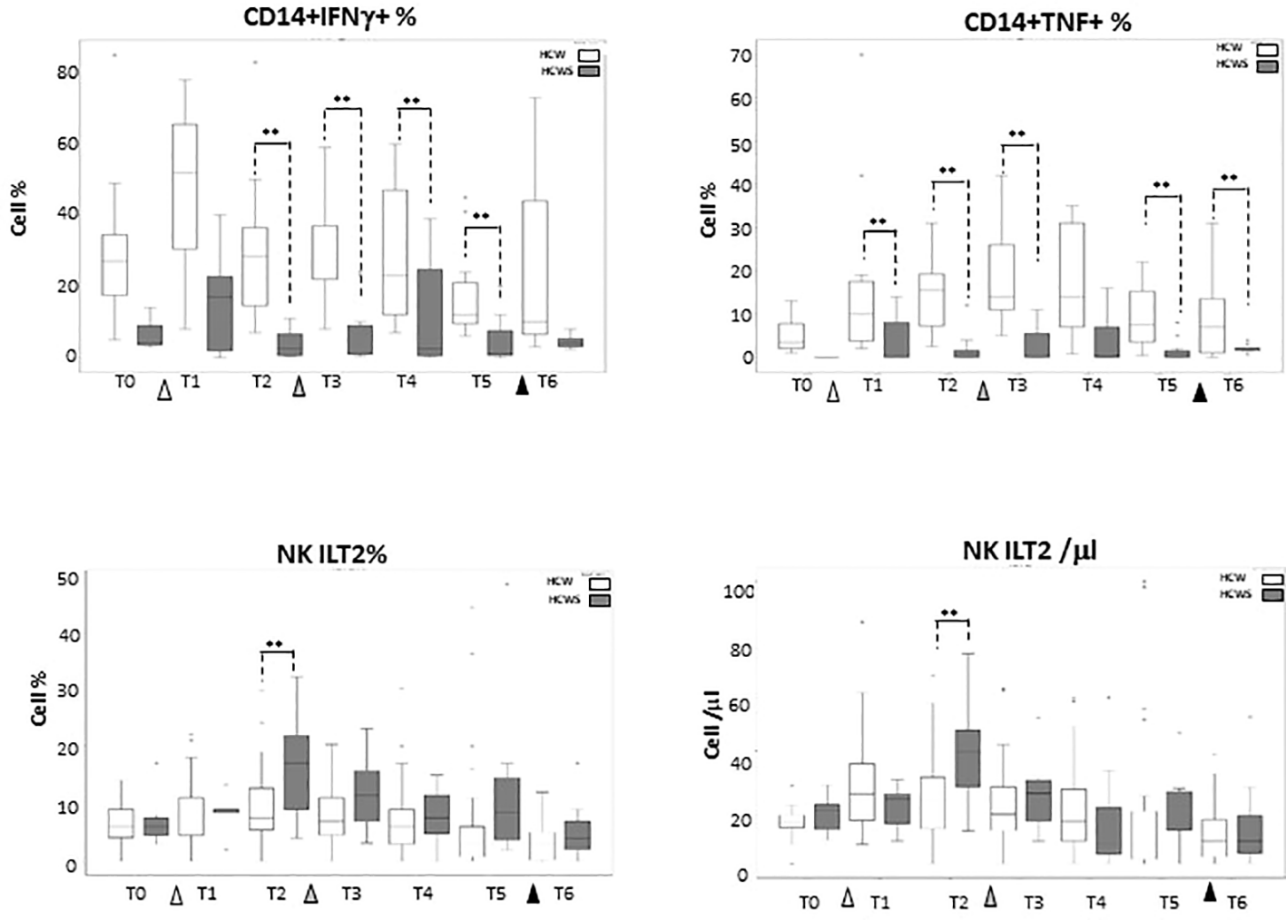
Innate Immune Responses stimulated by multiple doses of the BNT162b1 vaccine in individuals who had or had not been SARS-CoV-2-infected. IFN-γ-producing monocytes; TNF-producing monocytes and ILT-2-expressing NK cells in individuals who had not (HCW; white bars) or had (HCWS; black bars) been SARS-CoV-2-infected prior to the initiation of the vaccine protocol. Median values are shown at baseline (immediately before the first inoculation (white triangle) (T0), 7 (T1) and 21 (T2) days after initial inoculation, one (T3), three (T4) and six (T5) months after the first vaccine booster (grey triangle), and, finally, ten days after the second vaccine booster (black triangle) (T6). The boxes stretch from the 25th to the 75th percentile. The lines across the boxes indicate the median values. The lines stretching from the boxes indicate extreme values. Statistical significance is shown; **p<0,05.

Thirteen individuals who had been SARS-CoV-2-infected prior to receiving the first dose of the BNT162b1 vaccine were enclosed in the study as well; results obtained in these individuals (**Supplementary Tables 4**–**6**) were compared to those summarized above, stemming from analyses performed in subject who had never been SARS-CoV-2-infected. No statistically significant differences emerged when the effect of vaccination on monocytes subsets was compared between the two groups of individuals, with the exception of IFN-γ- and TNF-producing monocytes which were reduced at baseline in individuals who had previously been SARS-CoV-2 infected, and were not increased by the BNT162b1-vaccine. Notably, NK cells expressing the inhibitory receptor ILT-2, which were recently shown to be augmented in COVID-19 patients, were reduced by the second vaccine booster. These data are shown in **Figure 6**.

## Discussion

Within few months of sequencing the SARS-CoV-2 genome, a number of vaccines were developed and started being universally used to prevent infection. Amongst these vaccines, the ones based on the use of viral mRNA have proven to be associated with an extremely high rate of protection against COVID-19 which is secondary to their ability to elicit very potent humoral and cell mediated virus-specific immune responses (15, 16). The ability of SARS-CoV-2 mRNA vaccines to stimulate acquired immunity-based responses has been extensively analyzed (17–23), but very few data are available on the possible effect of these vaccines on innate immunity. We focused on this aspect and verified whether multiple doses of the BNT162b1 SARS-CoV-2 mRNA vaccine would modulate monocytes and natural killer (NK) cells, the two main populations that are responsible for innate immune responses to pathogens. Results herein show that this is indeed the case as vaccination induced long-lasting phenotypical and functional changes in both cell populations.

Periphery human monocyte cells can be divided into three main populations based on the expression of CD14 and CD16: 1) classical monocytes, characterized as CD14^high^, 2) intermediate monocytes which are CD14^high^CD16^dim^, and 3) non-classical monocytes, characterized as CD14^dim^/CD16^bright^ (24, 25). Results showed that vaccination significantly increased classical and intermediate, while reducing non-classical cells. Classical monocytes play an important role in tissue repair and in the elicitation of adaptive immune responses (25). They have finely honed phagocytic abilities and produce interleukin (IL)-6 and IL-8. Intermediate monocytes stimulate T cell proliferation and express high levels of surface markers involved in antigen-presenting cell–T cell interactions (25). Conversely, non-classical monocytes are characterized by an anti-inflammatory phenotype and respond poorly to TLR stimulation (26). Vaccine-associated increase of classical and intermediate monocytes is thus a positive phenomenon as it likely results in a prompter and more robust antiviral response and in an optimal ability to stimulate adaptive immune responses. Notably, these results are in agreement with recently published data showing that BNT162b2 vaccination induces a heightened innate immune response after secondary immunization relative to primary immunization, which includes an increase of intermediate monocytes (12).

NK cell subpopulations were differently affected by multiple doses of BNT162b1 as well. Thus, CD56^dim^, CD56 ^bright^, and CD56^dim^CD16^dim^ NK cells were increased whereas CD56^dim^CD16 ^bright^ NK cells were reduced by the vaccine. CD56^dim^ NK cells play an important role in antiviral host defense through cell-mediated cytotoxicity as they are enriched in perforin and produce high quantities of IFN-γ (27–29). CD56 ^bright^ and CD56^dim^CD16^dim^ cells contribute as well to antiviral immunity *via* the production of IFN-γ and TNF (30); CD56^dim^CD16^bright^ cells were instead shown to activate antibody-dependent cellular cytotoxicity (ADCC) in response to viruses (31). ADCC was recently suggested to contribute to viral control in COVID-19 patients, as antibodies elicited toward the SARS-CoV-2 S glycoprotein (S309- and S306) -transfected cells could efficiently trigger ADCC (32).

Notably, IFN-γ-producing monocytes and NK cells were significantly increased by vaccination. This observation is likely justified by the expansion of CD56 ^bright^ and CD56^dim^CD16^dim^ cells that was induced by the vaccine protocol. IFN-γ plays a fundamental role in initiating immune responses against intracellular pathogens and is pivotal in stimulating the generation of those TH1 CD4+ T lymphocytes that are the main actors in antiviral defense.

The ability of SARS-CoV-2 mRNA vaccines to elicit the generation of potent adaptive immunity-mediated antiviral responses has been repeatedly demonstrated (33–35), these data shed light on the initial immune events that are induced by vaccination and lead to the generation of a powerful, virus-specific cell-mediated immune response. Besides producing cytokines, NK cells are also characterized by the expression of stimulatory and inhibitory receptors; we observed that multiple doses of the BNT162b1 vaccine differentially affected the best characterized of these receptors. Hence, NK cells expressing the activation 2DS2 receptor were increased, whereas those expressing the inhibitory ILT-2 receptor were decreased by the vaccine protocol, indicating that the BNT162b1 vaccine gears NK-mediated immune responses toward activation. It is noteworthy to point out that very recent results show a reduction of CD56^dim^ and CD56^dim^CD16^bright^ and an increase of ILT-2- expressing NK cells in patients suffering from severe COVID-19 (36). With the exception of ADCC, thus, also in the case of NK cells, multiple vaccine doses seem to result in the optimal stimulation of innate immunity-mediated antiviral responses.

The net result of BNT162b1 vaccine-induced augmented IFN-γ production and increased expression of NK activator receptors is the activation of NK cells; these cells mediate lysis and apoptosis of infected targets *via* the release of perforin and granzymes, respectively. Results showed that this is exactly what happens upon vaccination. Thus, both granzyme- and perforin- containing NK were increased by vaccination indicating, once again, that the BNT162b1 mRNA vaccine is capable of optimally stimulating innate immunity-mediated antiviral mechanisms. Also in this case, whereas plentiful information is available on the ability of this vaccine to upregulate cytotoxic T cells (CTL)-mediated lysis of target cells (37–40), these are the first results showing that vaccination has the same effect on NK, a population of cytotoxic cells that, albeit being engaged in a different way, mediates the same effector functions of CTL.

Notably, the effect of the BNT162b1 mRNA vaccine on NK cells activation was also seen when individuals who underwent vaccination after having recovered from COVID-19 were analysed. Thus, recent results (36) indicate that the population of NK cells expressing the IL-T2 inhibitory receptor is increased in these patients, data presented here show that the second vaccine booster reduces such population, likely increasing NK cells activity. The effect of this vaccine on cytokine production by monocytes in previously infected individuals was different from that seen in individuals that had never been infected, as neither IFN-γ nor TNF were increased by vaccination in COVID-19-recovered subjects. This differs from the boosting effect on adaptive immune responses which is induced by vaccines in individuals who had been infected (41–46). These results are puzzling and need to be further investigated.

Arunachalam et al. demonstrated that the BNT162b2 vaccination in humans results in an enhanced innate immune response following a first booster dose (12). Our results expand those data and indicate that the net effect of BNT162b1 mRNA vaccination in individuals that had never suffered from COVID-19 is the activation and the phenotypical and functional optimization of the cell populations and molecules that are involved in innate immune responses. These effects are positively modulated by vaccine boosters and tend to be long lasting. Although a limitation of the present work is the sample size, results herein clarify the ability of this innovative vaccine to stimulate all the diverse components of the immune response and offer further support to the decision to proceed with the massive worldwide vaccination efforts to end the SARS-CoV-2 pandemic.

## Data availability statement

The raw data supporting the conclusions of this article will be made available by the authors, without undue reservation.

## Ethics statement

The studies involving human participants were reviewed and approved by Ethic committee of Fondazione don Carlo Gnocchi 06/17-02-2021. The patients/participants provided their written informed consent to participate in this study.

## Author contributions

MC designed the research and edited the manuscript; MS designed and performed the experiments and drafted the manuscript; FP, IM, AH, DT, and FL, performed experiments, analyzed results and created the figures; MI performed statistical analyses. All authors reviewed and approved the final manuscript.

## Funding

The study was supported by Italian Ministry of Health (Ricerca Corrente 2021).

## Conflict of Interest

The authors declare that the research was conducted in the absence of any commercial or financial relationships that could be construed as a potential conflict of interest.

## Publisher’s note

All claims expressed in this article are solely those of the authors and do not necessarily represent those of their affiliated organizations, or those of the publisher, the editors and the reviewers. Any product that may be evaluated in this article, or claim that may be made by its manufacturer, is not guaranteed or endorsed by the publisher.
