# Supplementary material for: Innate immune responses to three doses of the BNT162b2 mRNA SARS-CoV-2 vaccine
Source: Front Immunol. 2022 Aug 22;13:947320. doi: 10.3389/fimmu.2022.947320 (PMC9443429; doi:10.3389/fimmu.2022.947320)
Supplement: Supplementary file 1 [file Table_1.docx]

**Supplementary Table 1: Percentage of peripheral monocyte and Natural Killer (NK) subsets and of NK cells expressing the 2DS1, 2DS2 and 2DS4 activating or the 2DL1 and ILT2 inhibitory KIR receptors in receptors in 45 individuals who had never been SARS-CoV-2-infected and received three doses of the BNT162b1 mRNA vaccine at different time points: baseline (immediately before the first inoculation (T0), 7 (T1) and 21 (T2) days after initial inoculation, one (T3), three (T4) and six (T5) months after the first vaccine booster and ten days after the second vaccine booster (T6).Median , Interquartile range and significant differences are shown.**
