# Supplementary material for: Innate immune responses to three doses of the BNT162b2 mRNA SARS-CoV-2 vaccine
Source: Front Immunol. 2022 Aug 22;13:947320. doi: 10.3389/fimmu.2022.947320 (PMC9443429; doi:10.3389/fimmu.2022.947320)
Supplement: Supplementary file 3 [file Table_3.docx]

**Supplementary Table 3: Percentage of monocytes and NK cells producing cytokine and Perforin- or granzyme- containing and CD107a-expressing NK cells after spike-stimulation in Healthy Care Workers who had never been SARS-CoV-2-infected (HCW) and received three doses of the BNT162b1 mRNA vaccine at different time points: baseline (immediately before the first inoculation (T0), 7 (T1) and 21 (T2) days after initial inoculation, one (T3), three (T4) and six (T5) months after the first vaccine booster and ten days after the second vaccine booster (T6).Median , Interquartile range and significant differences are shown.**
