# Supplementary material for: Innate immune responses to three doses of the BNT162b2 mRNA SARS-CoV-2 vaccine
Source: Front Immunol. 2022 Aug 22;13:947320. doi: 10.3389/fimmu.2022.947320 (PMC9443429; doi:10.3389/fimmu.2022.947320)
Supplement: Supplementary file 4 [file Table_4.docx]

**Supplementary Table 4: Percentage of peripheral monocyte and Natural Killer (NK) subsets and of NK cells expressing the 2DS1, 2DS2 and 2DS4 activating or the 2DL1 and ILT2 inhibitory KIR in thirteen individuals who had been SARS-CoV-2-infected prior to receiving the first dose of the BNT162b1 vaccine at different time points: baseline (immediately before the first inoculation (T0), 7 (T1) and 21 (T2) days after initial inoculation, one (T3), three (T4) and six (T5) months after the first vaccine booster and ten days after the second vaccine booster (T6).Median , Interquartile range and significant differences are shown.**
